# Evaluation of ^99m^Tc-rhAnnexin V-128 SPECT/CT as a diagnostic tool for early stages of interstitial lung disease associated with systemic sclerosis

**DOI:** 10.1186/s13075-018-1681-1

**Published:** 2018-08-16

**Authors:** Janine Schniering, Li Guo, Matthias Brunner, Roger Schibli, Shuang Ye, Oliver Distler, Martin Béhé, Britta Maurer

**Affiliations:** 1 Center of Experimental Rheumatology, Department of Rheumatology, University Hospital Zurich, Gloriastrasse 25, 8091 Zurich, Switzerland; 2 Department of Rheumatology, Renji Hospital, Shanghai Jiao Tong University, Shanghai, China; 3 Center for Radiopharmaceutical Sciences, Villigen-PSI, Switzerland; 4 Institute of Pharmaceutical Sciences, Department of Chemistry and Applied Biosciences, Zurich, Switzerland

**Keywords:** Interstitial lung disease, Nuclear imaging, Apoptosis, Systemic sclerosis

## Abstract

**Background:**

Given the need for early detection of organ involvement in systemic sclerosis, we evaluated ^99m^Tc-rhAnnexin V-128 for the detection of early stages of interstitial lung disease (ILD) in respective animal models using single photon emission computed tomography (SPECT/CT).

**Methods:**

In bleomycin (BLM)-challenged mice, fos-related antigen 2 (Fra-2) transgenic (tg) mice and respective controls, lung injury was evaluated by analysis of hematoxylin and eosin (HE) and Sirius red staining, with semi-quantification of fibrosis by the Ashcroft score. Apoptotic cells were identified by TUNEL assay, cleaved caspase 3 staining and double staining with specific cell markers. To detect early stages of lung remodeling by visualization of apoptosis, mice were injected intravenously with ^99m^Tc-rhAnnexin V-128 and imaged by small animal SPECT/CT. For confirmation, biodistribution and ex vivo autoradiography studies were performed.

**Results:**

In BLM-induced lung fibrosis, inflammatory infiltrates occurred as early as day 3 with peak at day 7, whereas pulmonary fibrosis developed from day 7 and was most pronounced at day 21. In accordance, the number of apoptotic cells was highest at day 3 compared with saline controls and then decreased over time. Epithelial cells (E-cadherin+) and inflammatory cells (CD45+) were the primary cells undergoing apoptosis in the earliest remodeling stages of experimental ILD. This was also true in the pathophysiologically different Fra-2 tg mice, where apoptosis of CD45+ cells occurred in the inflammatory stage. In accordance with the findings on tissue level, at day 3 in the BLM and at week 16 in the Fra-2 tg model, biodistribution and/or ex vivo autoradiography showed increased pulmonary uptake of ^99m^Tc-rhAnnexin V-128 compared with controls. However, accumulation of the radiotracer and thus the signal intensity in lungs was too low to allow the differentiation of healthy and injured lungs in vivo*.*

**Conclusion:**

At the tissue level, ^99m^Tc-rhAnnexin V-128 successfully demonstrated early stages of ILD in two animal models by detection of apoptotic epithelial and/or inflammatory cells. In vivo, however, we did not detect early lung injury. It remains to be investigated whether the same applies to human ILD.

**Electronic supplementary material:**

The online version of this article (10.1186/s13075-018-1681-1) contains supplementary material, which is available to authorized users.

## Background

Systemic sclerosis (SSc) is a devastating multisystem autoimmune connective tissue disease with lung involvement as the primary cause of death [1]. Interstitial lung disease (ILD) occurs early in the disease course and affects 40–70% of patients. Diagnostic tools such as pulmonary function tests (PFTs) or high-resolution computed tomography (HRCT) often only detect irreversibly compromised lung function and structure [2]. Consequently, there is a need for the diagnosis of early, potentially reversible disease stages. This need could be met by nuclear medicine applications such as single photon emission computed tomography (SPECT/CT) and positron emission tomography (PET). These highly sensitive and specific methodologies allow the real-life visualization of pathophysiological processes and have become valuable diagnostic tools in oncology [3].

In SSc-ILD, pulmonary damage at its early stages is characterized by apoptosis of epithelial cells (EPC) (up to 80%) [4] and inflammatory cells caused by, for example, cigarette smoke, infections, environmental exposures or micro-aspiration and/or locally increased oxidative and endoplasmatic reticulum stress [5, 6]. Notably, in experimental animal models of ILD, EPC damage [7] or the delivery of apoptotic cells induce lung fibrosis [8, 9], whereas blocking of apoptotic pathways prevents or attenuates the development [10–12]. Dying cells release cellular contents such as adenosine triphosphate (ATP), uric acid or high-mobility group protein B1 (HMGB-1), some of which being recognized as danger-associated pathogens (DAMPs) [13, 14]. In experimental ILD, signaling via danger receptors, including, for example, toll like receptors (TLRs), initiates innate immune responses, thereby promoting inflammation and fibrosis mainly through the NFkB/inflammasome and IL-1 pathways [15]. In physiologic conditions, the DAMP-mediated influx of inflammatory cells leads to the clearance of apoptotic debris and the resolution of inflammation. In ILD, probably due to a genetic predisposition, exaggerated DAMP signaling occurs with a sustained pro-inflammatory response, part of which is attributed to inefficient phagocytosis of apoptotic cell debris (= efferocytosis) [16, 17]. Among the phagocytosing cells, alternatively activated macrophages, which are predominant in ILD [18, 19], are a major source of transforming growth factor (TGF)β [20]. TGFβ induces apoptosis of EPCs, thereby further enhancing the loss of functional epithelium [21, 22]. Furthermore, TGFβ mediates the differentiation of fibroblasts into myofibroblasts rendering them resistant to apoptosis [23]. This results in massively increased and perpetuated secretion of extracellular matrix proteins. Although less well-investigated, it has been suggested that adaptive immune responses might also be involved in pathogenesis in ILD. Potential mechanisms include cross-presentation of cellular DAMPs from apoptotic epithelial or inflammatory cells by, for example, dendritic cells, which could drive the activity of cytotoxic T cells and thereby increase lung damage [24, 25]. In addition, patient-derived data and data derived from experimental ILD suggest a potential pathogenic involvement of B cells [25–27] and a propensity towards a T helper 2 (Th2) response [28]. Overall, in ILD, there is a vicious cycle of dysregulated pro-apoptotic and anti-apoptotic mechanisms involving different cell types, which identifies apoptosis as an important initiator and driver of lung fibrosis.

One of the first signals of cells undergoing apoptosis is the rapid redistribution of phosphatidylserine (PS) onto the cell surface, where annexin V binds with high affinity. PS constitutes 10–15% of the phospholipids of the inner leaflet of the plasma membrane [29]. Upon the onset of apoptosis, closely following activation of caspase 3, translocation of PS onto the cell surface results in a 100–1000-fold increase of annexin V binding sites per cell [30]. Notably, annexin V may also identify necrotic cells, since the disruption of the membrane of necrotic cells may allow binding of annexin V to PS at the inner leaflet [31]. In human pilot studies, technetium-99 m (^99m^Tc)-labeled annexin V has been used to detect apoptosis and necrosis in the context of acute myocardial infarction [32] and cardiac allograft rejection [33]. Recent data from animal studies using models of (infectious) endocarditis [34], atherosclerosis [35], myocarditis [36] and rheumatoid arthritis [37] suggest a potential use for the detection of early inflammatory disease stages.

In this study, we aimed to evaluate the potential of ^99m^Tc-rhAnnexin V-128-based SPECT/CT to visualize early stages of lung remodeling in two representative mouse models of SSc-ILD, the model of bleomycin (BLM)-induced lung fibrosis [38] and the Fos-related antigen 2 (Fra-2) transgenic (tg) mouse model [39, 40].

## Methods

### Animal experiments

All animal experiments were approved by the cantonal authorities and performed according to the Swiss animal welfare guidelines. For all experiments, mice were randomly assigned into the different study groups.

### Model of BLM-induced lung fibrosis

The BLM-induced lung fibrosis model is a commonly used animal model to study pulmonary inflammation and fibrosis mimicking SSc-related ILD. BLM-induced ILD develops in a time-dependent manner with inflammation occurring by day 3 and peaking at day 7, while pulmonary fibrosis develops later starting at day 7 and getting maximal at day 21 after the BLM administration [41]. To induce lung inflammation and fibrosis, female C57Bl6/J mice age 7–8 weeks (Janvier Labs, Le Genest-Saint-Isle, France) were intratracheally instilled with bleomycin sulfate (Baxter, Kantonsapotheke Zurich, Switzerland) at a dosage of 4 U/kg of body weight. Control mice received equivalent volumes of 0.9% saline solution. Mice were sacrificed at days 3, 7, 14 and 21 after the instillation of BLM (*n* = 3–4 receiving saline, *n* = 3–9 receiving BLM).

### Fra-2 tg mouse model

Fra-2 tg mice express the transcription factor Fra-2 of the activator protein-1 family under the control of the ubiquitous major histocompatibility complex class I antigen H2Kb promotor [39]. Fra-2 tg mice develop a multi-organ phenotype, most importantly affecting the skin [40, 42] and the lungs [43, 44]. Lung involvement of Fra-2 tg mice is characterized by non-specific interstitial pneumonia with mild interstitial fibrosis and severe proliferative vascular remodeling resembling pulmonary hypertension [43, 44]. Fra-2 tg mice were newly generated and provided by Sanofi Genzyme (Framingham, MA, USA) and backcrossed from a mixed genetic background (C57Bl6/J × CBA) to a pure C57Bl6/J background for more than 10 generations. In this study, 10, 14 and 16 week-old female Fra-2 tg mice (*n* = 2–6) were used. Wild-type littermates (*n* = 2–4) served as controls.

### Histological analysis

For histological analysis, lungs were transcardially perfused with sterile phosphate-buffered saline solution (PBS) to remove residual blood, fixed with 10% neutral-buffered formalin and embedded into paraffin. Lung sections (4 μm thick) were stained with hematoxylin and eosin (HE) for the assessment of the overall lung architecture and with Sirius red for the visualization of collagen deposition according to standard protocols. For the analysis of lung fibrosis, the semi-quantitative Ashcroft score was applied as described previously [45]. In brief, successive fields within the lung sections stained with Sirius red to identify fibrotic areas (red) were observed under a microscope at × 100 magnification and allotted a score from 0 (normal) to 8 (total fibrosis) according to the severity (Table 1).

**Table 1:** Fibrotic lung remodeling according to the Ashcroft score [22]

Grade of fibrosis	Histological changes
0	Normal lung
1	Minimal fibrous thickening of alveolar/bronchial walls
2	*Intermediary stage between 1 and 3*
3	Moderate thickening of walls without obvious damage to the lung architecture
4	*Intermediary stage between 3 and 5*
5	Increased fibrosis with definite damage to lung structure and formation of fibrous bands or small fibrous masses
6	*Intermediary stage between 5 and 7*
7	Severe distortion of structure and large fibrous areas; honeycombing lung is placed in this category
8	Total fibrous obliteration of the field

All histological specimens were evaluated by at least two experienced examiners in a blinded fashion. The mean of their individual scores was considered the final fibrotic score. Staining was recorded automatically by the AxioScan.Z1 slidescanner (Carl Zeiss, Feldbach, Switzerland) using a Plan-Apochromat 20×/0.8 M27 objective.

### TUNEL assay

To detect apoptotic and necrotic cells in paraffin-embedded lung sections, terminal deoxynucleotidyl transferase (TdT)-mediated dUTP nick end labeling (TUNEL) [46] was performed applying the ApopTag® Fluorescein in Situ Apoptosis Detection Kit (Millipore, USA) according to the manufacturer’s instructions. In brief, after deparaffinization and rehydration, sections were treated with proteinase K (20 μg/mL) for 15 min at room temperature (RT). Subsequently, equilibration buffer was applied for ~ 10 s, and then the specimens were incubated for 1 h in working strength TdT enzyme solution at 37 °C. Following incubation in stop/wash buffer for 10 min to terminate the reaction, sections were incubated for 30 min in working strength anti-digoxigenin conjugate at RT in the dark to visualize the DNA fragments. Finally, slides were counterstained with 0.5 μg/mL 4',6-diamidino-2-phenylindole (DAPI) and mounted with fluorescence mounting medium. Sections treated only with reaction buffer, but without TdT enzyme were used as negative controls. To quantify the numbers of apoptotic cells, pictures of six randomly chosen high power fields (HPFs)/slide at × 200 magnification were taken by a blinded examiner using a wide-field fluorescence microscope (Olympus BX53, Volketswil, Switzerland). TUNEL+ nuclei were quantified by automatic counting using Image J (NIH version1.47 t).

### Immunohistochemical assessment

To visualize specifically apoptotic cells in paraffin-embedded lung sections, we performed immunohistochemical assessment for cleaved caspase 3. In brief, after deparaffinization and rehydration, antigen was retrieved using 10 mM sodium citrate buffer (pH = 6.0) at 95 °C for 15 min. After blocking endogenous peroxidase activity with 3% hydrogen peroxide for 15 min at RT, sections were treated with 10% normal goat serum (1 h, RT) to prevent unspecific antibody binding and blocked for endogenous biotin using an Avidin/Biotin blocking kit (Vector Laboratories, Burlingame, CA, USA). Afterwards, specimens were incubated with monoclonal rabbit anti-mouse cleaved caspase 3 (1:1000, clone 5A1E, Cell Signaling, USA) overnight at 4 °C. Isotype-matched and concentration-matched IgG was used as a negative control. Afterwards, a biotin-labeled goat anti-rabbit secondary antibody (Vector Laboratories) was applied (1:200, 30 min, RT). This was followed by incubation with the Vectastain ABC Elite HRP kit (Vector Laboratories, 30 min, RT). Finally, staining was visualized using 3,3′-diaminobenzidine (DAB, Vector Laboratories) and counterstained with methyl green.

Staining was recorded automatically by the Zeiss AxioScan.Z1. slidescanner using a Plan-Apochromat 20×/0.8 M27 objective. For cell counting, six randomly selected, non-overlapping HPFs at × 400 magnification were extracted per sample using the Zen 2.0 lite (blue edition) software. All analyses were performed by two blinded examiners.

### Immunohistochemical double staining

To identify the cell types undergoing apoptosis, immunohistochemical double staining with cell-type-specific markers were performed. For the double staining, cleaved caspase 3-stained lung sections (without counterstain) were subjected to an additional heat-mediated antigen retrieval step using 10 mM sodium citrate buffer to avoid unspecific staining when using primary antibodies originating from the same species. After repetition of the aforementioned blocking steps, the following primary antibodies were added: monoclonal mouse anti-mouse alpha smooth muscle actin (αSMA, 1:750, clone 1A4, Sigma, Switzerland), monoclonal rat anti-mouse CD45 (1:50, clone 30-F11, BD Pharmingen, San Jose, CA, USA), polyclonal rabbit anti-mouse von Willebrand factor (vWF) (1:100, abcam, Cambridge, UK), and monoclonal mouse anti-mouse E-cadherin (1:400 clone M168, abcam). Isotype-matched and concentration-matched IgGs were used as negative controls. All primary antibodies were incubated overnight at 4 °C except for αSMA with a 1 h incubation time at RT. Next, a direct alkaline phosphatase-labeled goat anti-mouse secondary antibody (Dako, Baar, Switzerland), or biotin-labeled goat anti-rat, anti-mouse, or anti-rabbit secondary antibodies (all from Vector Laboratories) were applied on the sections (30 min, RT). This was followed in the latter case by incubation with the Vectastain ABC Elite HRP kit. Finally, staining was developed using Vector Red (Vector Laboratories) or HistoGreen (Histoprime; Linaris, Wertheim-Bettingen, Germany).

Pictures were recorded at × 400 magnification using the Olympus BX53 microscope in brightfield mode. For semi-quantification of the number of apoptotic leucocytes, epithelial cells, myofibroblasts or endothelial cells, three randomly selected HPFs were taken per sample and double positive cells were manually counted by two blinded examiners.

### Biodistribution of ^99m^Tc-rhAnnexin V-128

After intravenous (i.v.) injection of ~ 10 MBq ^99m^Tc-rhAnnexin V-128 (kindly provided by Advanced Accelerator Applications, Novartis Company, Saint-Genis Pouilly, France) ex vivo biodistribution studies were performed in BLM-treated mice and saline treated controls at day 3 after the BLM instillation (*n* = 3, each) to assess the radiotracer uptake in the organs and tissues of interest. Mice were killed using carbon dioxide 4 h post injection (p.i.) of the radiotracer and blood was taken, and organs of interest were harvested and weighed. Radioactivity counts were measured in a γ-counter (Packard Cobra II Auto Gamma, Perkin Elmer, Switzerland). The percentage of injected activity per gram tissue (% IA/g) was calculated for each sample.

### Ex vivo autoradiography

After i.v. injection of the radiotracer ^99m^Tc-rhAnnexin V-128 (~ 10 MBq) (1 h p.i. Fra-2 model, 4 h p.i. BLM model), lungs were harvested and embedded in Tissue-Tek O.C.T. compound, and snap frozen at optimal cutting temperature. Fresh frozen sections 8-μm thick were cut using a cryotom and were subsequently exposed on a phosphoimager screen (super resolution type SR, PerkinElmer, Waltham, USA) for 30 min. The phosphoimager screen images were read using a CyclonePlus (PerkinElmer, Waltham, USA).

### In vivo imaging using small animal SPECT/CT

BLM-treated, Fra-2 tg mice and respective controls were scanned using a small animal SPECT/CT scanner (NanoSPECT/CT, Mediso, Budapest, Hungary) at 4 h after injection of ~ 10 MBq ^99m^Tc-rhAnnexin V-128 [36, 47]. Imaging were acquired using the Nucline software (version 1.02, Bioscan). SPECT/CT data were reconstructed iteratively by HiSPECT software (version 1.4.3049, Scivis GmbH, Göttingen, Germany) using ^99m^Tc γ-energies of 140 keV ± 10%, and visualized with VivoQuant (version 3.0, Invicro, Boston, USA).

### Statistical analysis

Statistical analysis was performed using GraphPad Prism (version 7.02, GraphPad Software, La Jolla, CA USA). Non-parametric and non-related data were expressed as median ± min/max values and the Mann-Whitney U test was applied. For parametric, non-related data, expressed as mean ± standard deviation (SD), the unpaired *t* test was performed. *P* values less than 0.05 were considered statistically significant.

## Results

### Apoptosis is an early phenomenon in the development of lung fibrosis in different murine ILD models

**Fig. 1:**
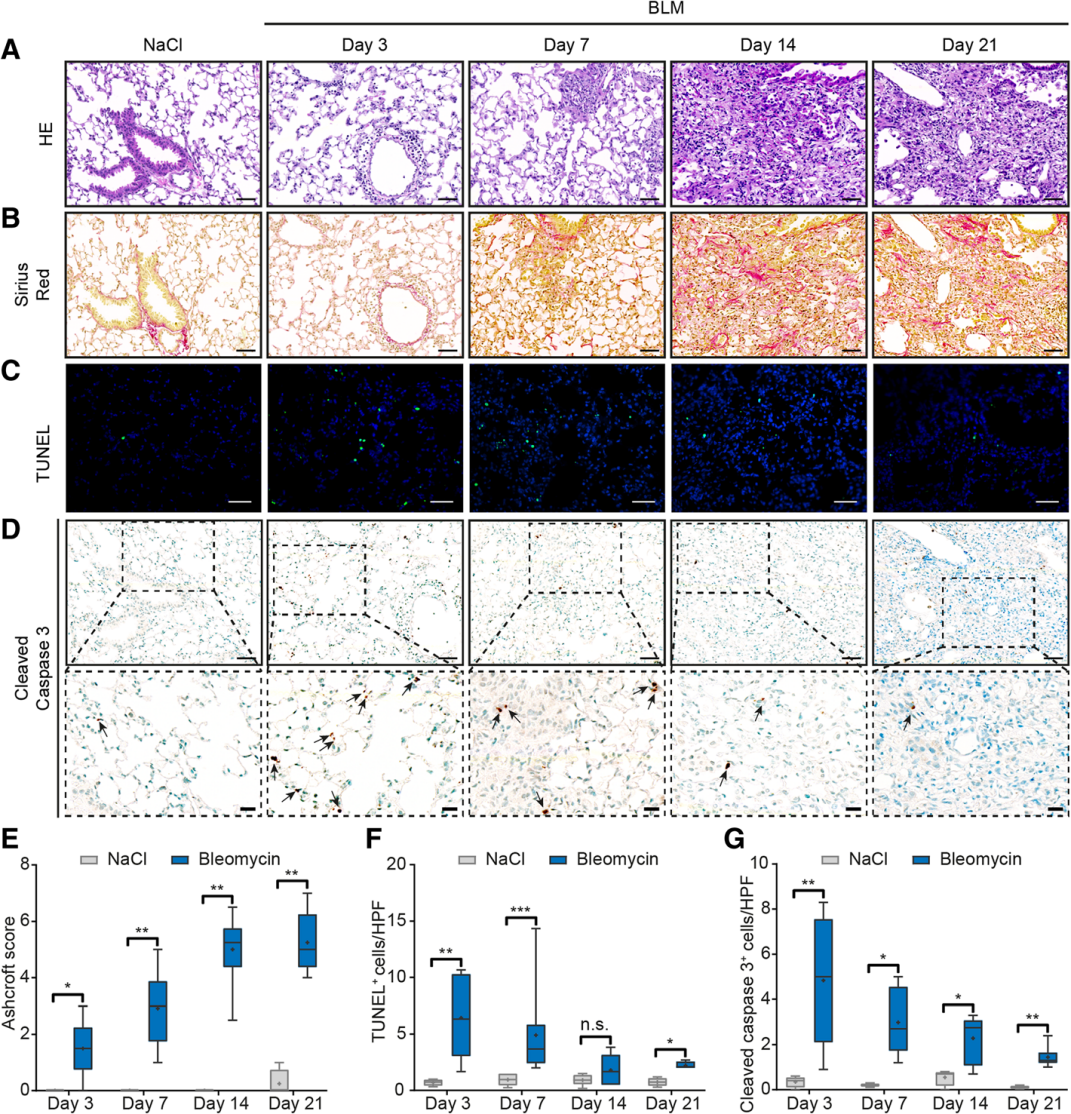
Time line of apoptosis in the model of bleomycin (BLM)-induced lung fibrosis. Hematoxylin and eosin (HE) staining (× 200) (**a**); Sirius Red staining (collagen fibers identified by red staining; × 200) (**b**); TUNEL staining (× 200) (**c**); cleaved caspase 3 staining (× 200) (**d**), inlets show higher magnifications (× 400), arrows highlight apoptotic cells; Ashcroft scores (**e**); semi-quantification of TUNEL+ cells (**f**) and semi-quantification of cleaved caspase 3+ cells (**g**): *n* = 4 (saline) or *n* = 6–9 (BLM). Data in box plots are expressed as median (line), mean (+) and minimum and maximum values: *p < 0.05, **p < 0.01, ***p < 0.001, Mann-Whitney U test. Scale bars 50 μm and 20 μm for inlets

**Fig. 2:**
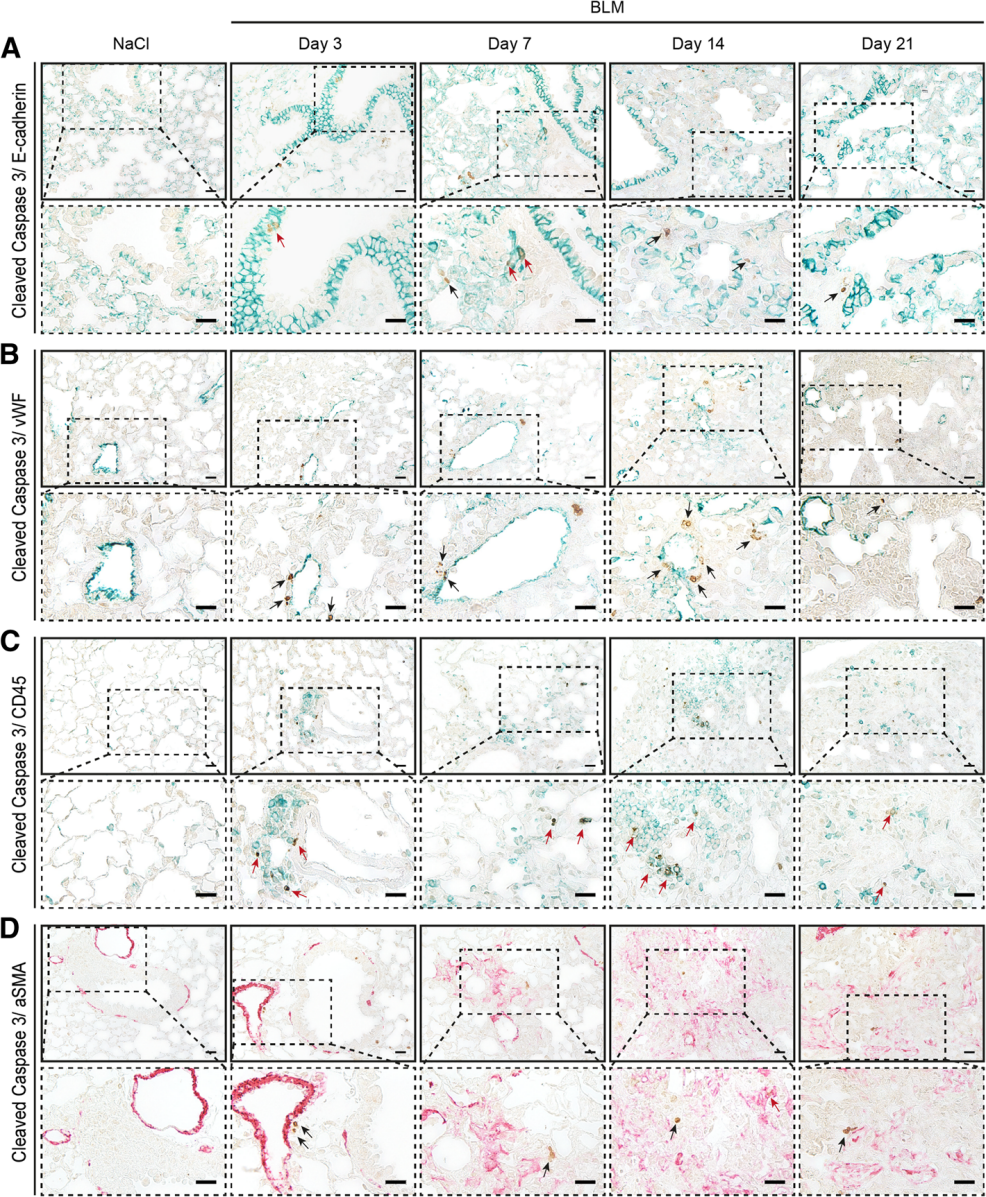
Identification of cell types undergoing apoptosis in the model of bleomycin (BLM)-induced lung fibrosis. Immunohistochemical co-staining of cleaved caspase 3 (brown) with the epithelial cell marker E-cadherin (green) (**a**), with the endothelial cell marker von Willebrand factor (vWF) (green) (**b**), with the pan-leucocyte marker CD45 (green) (**c**) and with the smooth muscle cell and myofibroblast marker alpha smooth muscle actin (αSMA) (red) (**d**). Magnification is × 400. Inlets represent zoomed images. Representative pictures from three mice each are shown. Scale bars 20 μm. Red arrows highlight double staining with the cell-type-specific markers, black arrows show single stained apoptotic cells

Upon BLM challenge, lung remodeling occurred over time with influx of mononuclear cells (days 3–7), loss of alveoli and thickening of the interstitium (days 7–21) as assessed by HE staining (Fig. 1a). Fibrosis, i.e. the deposition of extracellular matrix proteins as visualized by Sirius Red staining (Fig. 1b) followed the inflammatory stage (days 3–7) and was most pronounced at days 14 and 21, which was also reflected in the semi-quantitative Ashcroft score (median_(Q1,Q3)_ Ashcroft score at day 21 = 5_(4.4, 6.3)_, *p* = 0.0286; Fig. 1e).To reliably detect apoptosis ex vivo, we performed TUNEL as well as caspase 3 stainings. There was a significant increase in TUNEL+ apoptotic cells as early as day 3 (median_(Q1,Q3)_ = 6.34 _(3.04,10.3)_ versus 0.75_(0.42,0.96)_, *p* = 0.0095; Fig. 1c), which was confirmed by staining for cleaved caspase 3 (Fig. 1d). Semi-quantification showed a rapid decline of apoptotic cells after day 7 (Fig. 1f, g), at which inflammation subsided and fibrosis developed (Fig. 1a, /b). In accordance with the pathophysiology of BLM-induced lung injury, co-staining with specific cell markers identified the apoptotic cells (cleaved caspase 3+) at days 3, 7, 14 and 21 as EPC (E-cadherin+; Fig. 2a) and leucocytes (CD45+; Fig. 2c) (Additional file 1). At the given time points, apoptosis of endothelial cells (vWF+; Fig. 2b) or fibroblasts (αSMA+; Fig. 2d) did not occur or very rarely occurred (Additional file 1). In summary, in the investigated time period ranging from days 3 to 21, apoptosis peaked at day 3. Then, it decreased rapidly until day 7 and thereafter gradually until day 21, although the numbers of apoptotic cells still remained higher in the lungs of BLM-challenged mice compared to controls (Fig. 1f, g).

**Fig. 3:**
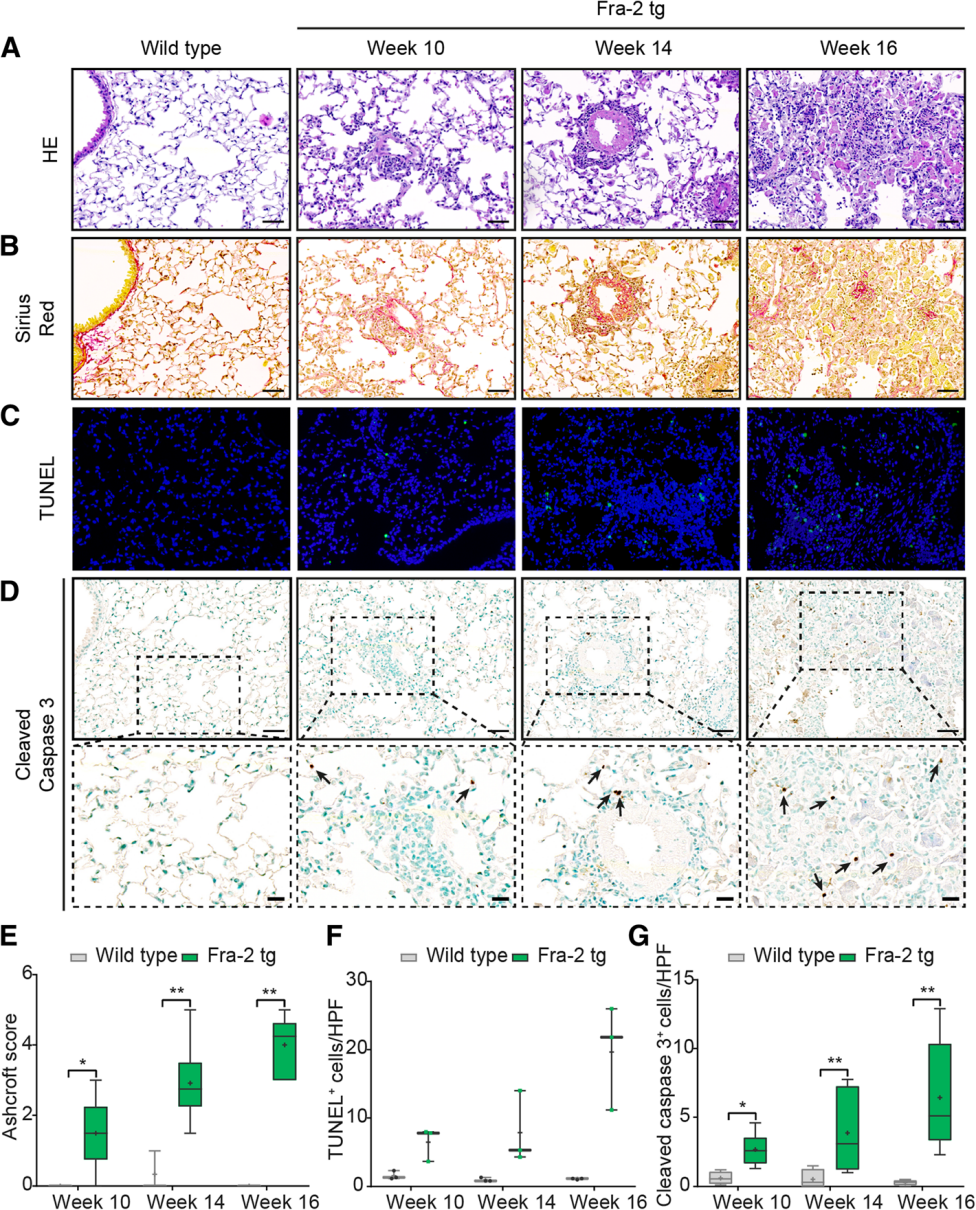
Time line of apoptosis in the fos-related antigen 2 (Fra-2) transgenic (tg) mouse model. Hematoxylin and eosin (HE) staining (× 20) (**a**); Sirius Red staining (collagen fibers identified by red staining; × 200) (**b**); TUNEL staining (× 200) (**c**); cleaved caspase 3 staining (× 200) (**d**), inlets show higher magnifications (×400), arrows highlight apoptotic cells; Ashcroft scores (**e**), semi-quantification of TUNEL+ cells (**f**) and semi-quantification of cleaved caspase 3+ cells (**g**): *n* = 3–4 (wild type) or n = 3–6 (Fra-2 tg). Data in box plots are median (line), mean (+) and minimum and maximum values: *p <0.05, **p < 0.01, Mann-Whitney U test. Scale bars 50 μm and 20 μm for inlets

**Fig. 4:**
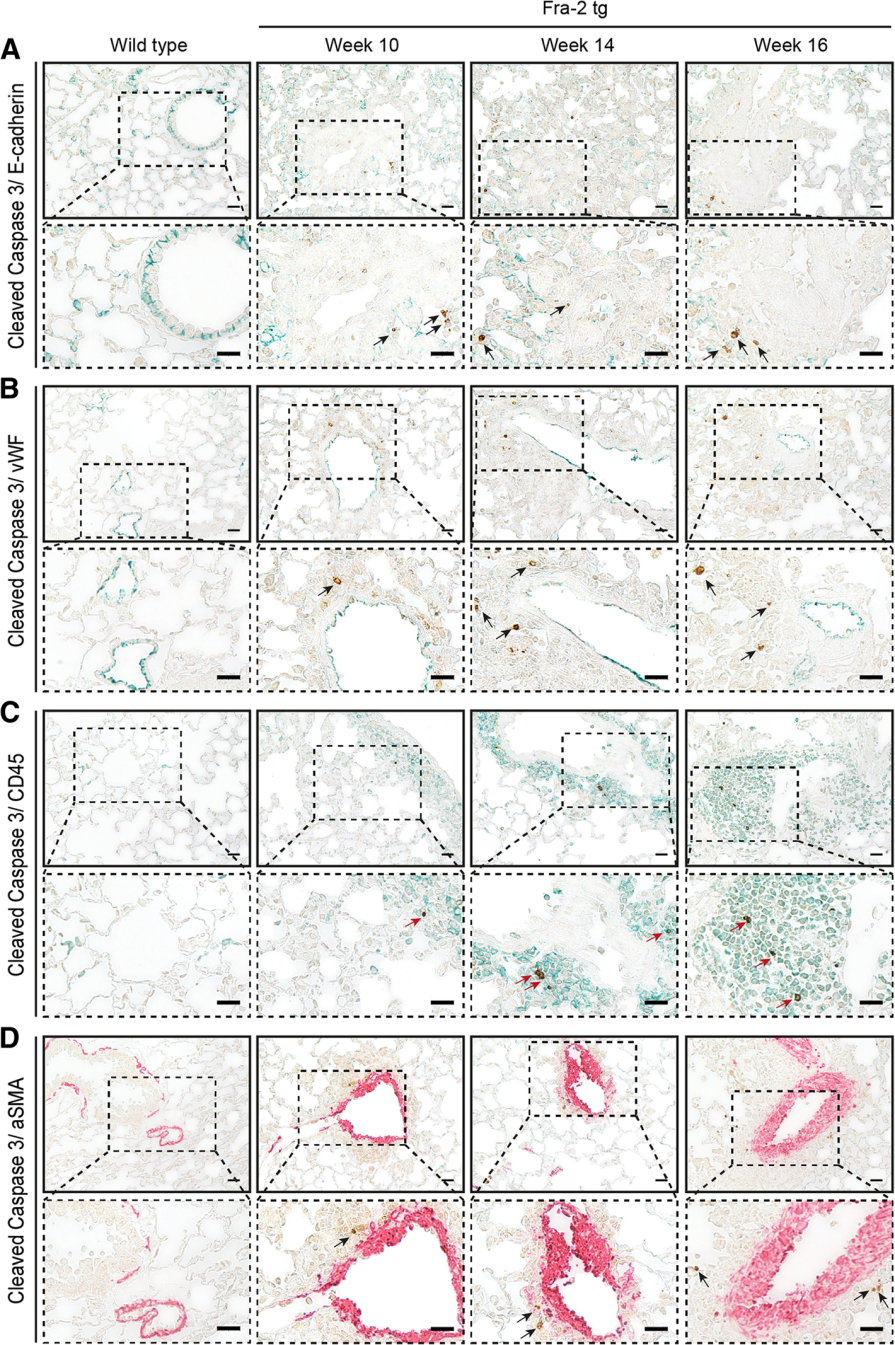
Identification of cell types undergoing apoptosis in the fos-related antigen 2 (Fra-2) transgenic (tg) mouse model. Immunohistochemical co-staining of cleaved caspase 3 (brown) with the epithelial cell marker E-cadherin (green) (**a**), with the endothelial cell marker von Willebrand factor (vWF) (green) (**b**), with the pan-leucocyte marker CD45 (green) (**c**), and with the smooth muscle cell and myofibroblast marker alpha smooth muscle actin (αSMA) (red) (**d**). Magnification is × 400. Inlets represent zoomed images. Representative pictures from three mice each are shown. Scale bars 20 μm. Red arrows highlight double staining with the cell-type-specific markers, black arrows show single stained apoptotic cells

In contrast to the model of BLM-induced lung fibrosis, in which - following the route of administration - lung injury started peribronchially and then spread to the interstitium (Fig. 1), in Fra-2 tg mice, and pulmonary vasculopathy was the initial pathophysiologic event starting from week 10 as shown by HE staining (Fig. 3a). Later, perivascular inflammation (week 14), then interstitial inflammation and to a lesser extent fibrosis (week 16), visualized by Sirius Red staining, occurred (Fig. 3b). With more inflammation in the lungs of Fra-2 tg mice, yet less fibrosis compared to BLM-challenged mice, the semi-quantitative Ashcroft score at the peak of lung remodeling in Fra-2 tg mice (week 16) (median_(Q1,Q3)_ = 4.3 _(3.0, 4.6)_, *p* = 0.0095; Fig. 3e) was not as high as in the respective period of the BLM lung model (day 14) (median_(Q1,Q3)_ = 5.3 _(4.4, 5.8)_, *p* = 0.0048; Fig. 1e). In line with the different pathophysiology, during the period of observation (weeks 10–16) we observed a time-dependent increase in pulmonary apoptotic cells (TUNEL+, Fig. 3c; cleaved caspase 3+, Fig. 3d) starting from week 10, continuing in week 14 and reaching its peak at week 16 as assessed semi-quantitatively (Figs. 3f, g). Compared with the BLM-challenged mice, co-staining with the respective cell markers (Figs. 4a–d) showed that CD45+ (Fig. 4c) leucocytes accounted for the clear majority of apoptotic cells in the lungs (Additional file 2).

### Evaluation of the potential of ^99m^Tc-rhAnnexin V-128 to visualize apoptosis in different animal models of ILD

**Fig. 5:**
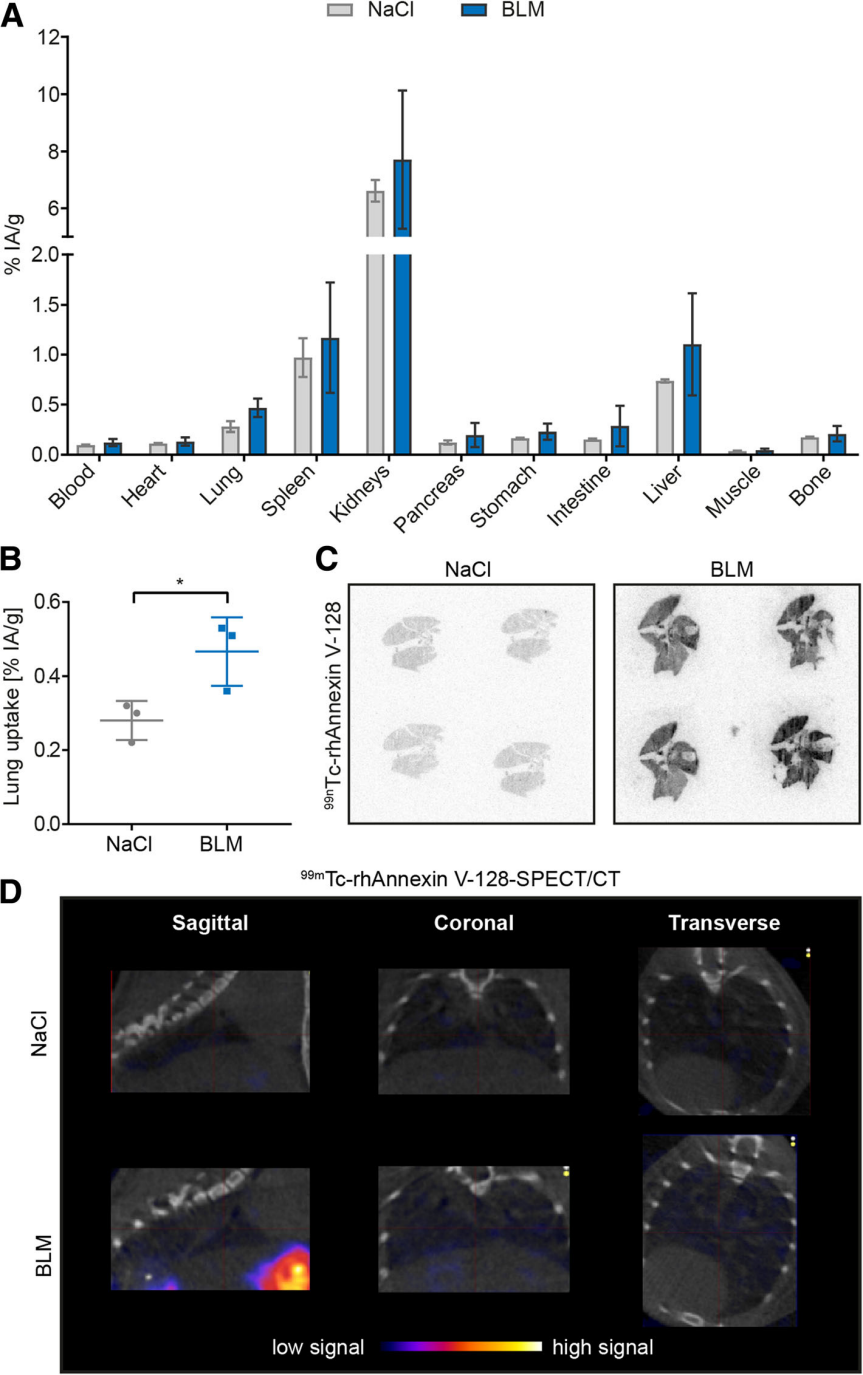
Imaging of apoptotic cells with ^99m^Tc-rhAnnexin V-128 in bleomycin (BLM)-treated mice. **a** Biodistribution of ^99m^Tc-rhAnnexin V-128 in relevant organs of BLM-treated mice and saline controls. **b** Significantly greater lung uptake of the ^99m^Tc-rhAnnexin V-128 radiotracer (4 h post injection (p.i.)) in the lungs of BLM-treated mice at day 3 after the BLM instillation. **c** Ex vivo autoradiography of frozen lung sections derived from BLM-treated mice and controls showing higher accumulation of ^99m^Tc-rhAnnexin V-128 (4 h p.i.) in BLM-treated mice versus controls at day 3 post instillation. **d** In vivo single photon emission computed tomography (SPECT/CT) of ^99m^Tc-rhAnnexin V-128 (4 h p.i.) administrated to BLM-treated mice and saline controls at day 3 post instillation. Herein, the chest cavity including the heart and the lungs is shown. Data are expressed as mean ± SD, *n* = 3, *p < 0.05, unpaired parametric Student’s *t* test. % IA/g, percentage of injected activity per gram tissue

**Fig. 6:**
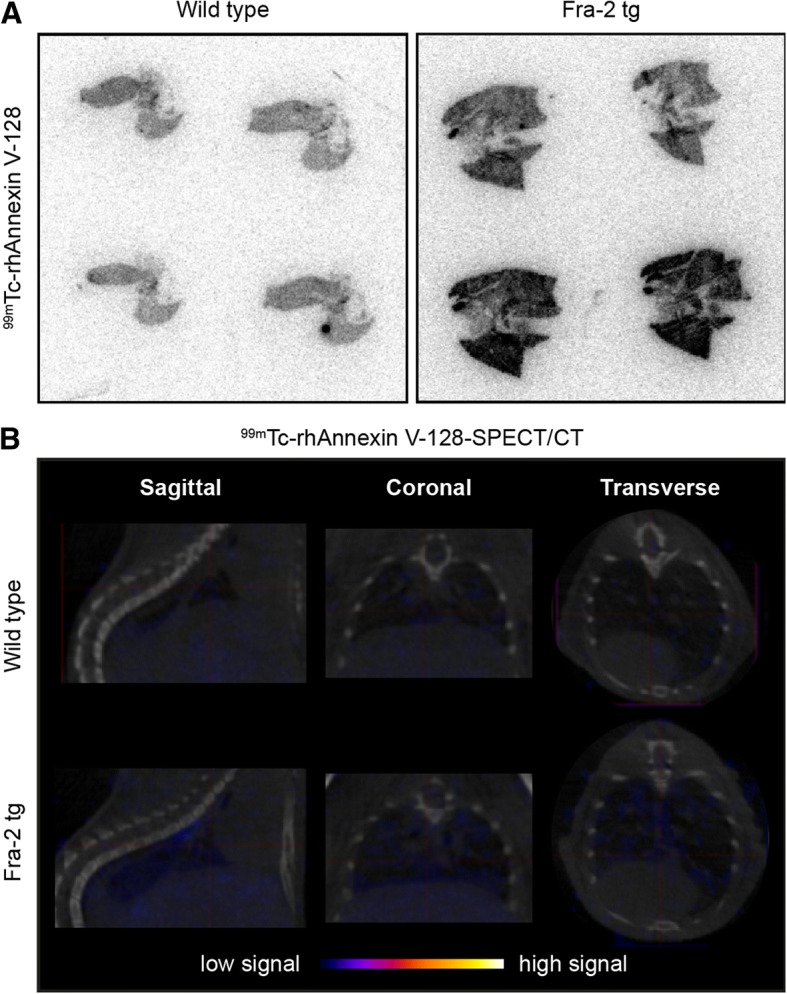
Imaging of apoptotic cells with ^99m^Tc-rhAnnexin V-128 in fos-related antigen 2 (Fra-2) transgenic (tg) mice. **a** Ex vivo autoradiography for ^99m^Tc-rhAnnexin V-128 in the lungs of Fra-2 tg mice versus wild-type mice. Frozen lung sections derived from a Fra-2-tg mouse and a wild-type mouse showed higher accumulation of ^99m^Tc-rhAnnexin V-128 (1 h post injection (p.i.)) in lungs of transgenic mice. **b** In vivo single photon emission computed tomography (SPECT/CT) of ^99m^Tc-rhAnnexin V-128 (4 h p.i.) administrated to Fra-2 tg mice and wild-type littermates at age 19 weeks. Herein, the chest cavity including the heart and the lungs is shown

Following our observation at the tissue level that apoptosis could serve as a surrogate marker for early lung remodeling, we next evaluated the diagnostic potential of the radiotracer ^99m^Tc-rhAnnexin V-128 in both animal models of experimental ILD. In the BLM lung model, at day 3, biodistribution analysis of ^99m^Tc-rhAnnexin V-128 revealed a significant difference in the radiotracer uptake (% IA/g) only in the lungs of BLM-treated mice compared to controls (mean ± SD = 0.47 ± 0.09% IA/g versus 0.28 ± 0.05% IA/g; Fig. 5b), yet not in other organs (Fig. 5a). These findings were confirmed by ex vivo autoradiography of frozen lung sections, where a higher accumulation of ^99m^Tc-rhAnnexin V-128 (4 h p.i.) was observed in BLM-treated mice compared with controls at day 3 post-instillation (Fig. 5c). In the Fra-2 tg mouse model, ^99m^Tc-rhAnnexin V-128 clearly demonstrated apoptosis in the inflamed lungs of Fra-2 tg mice using ex vivo autoradiography (1 h p.i.; Fig. 6a).

Despite the encouraging ex vivo results, in vivo imaging of the earliest lung remodeling by visualization of apoptosis with ^99m^Tc-rhAnnexin V-128 SPECT/CT, in the applied experimental conditions, was not successful (Figs. 5d, 6b). No specific pulmonary accumulation of the radiotracer was observed in either healthy or diseased mice (4 h after injection of the radiotracer).

## Discussion

The impending approval of molecular targeted, disease-specific therapies in SSc will provide unique treatment opportunities [48]. However, to improve patient outcome effectively, there is a need for earlier diagnosis of organ involvement to create a real window of opportunity. Sensitive nuclear imaging methodologies allowing the visualization of pathophysiologic processes in real time have become an integral part of the management of patients with cancer [3]. Although routinely available, the use of ^18^F-FDG-PET for the diagnosis and monitoring of inflammatory diseases is limited. In inflammatory conditions, increased signal intensity due to metabolic cell activity may reflect both developing and resolving tissue remodeling and is therefore not selective for early inflammatory stages [35, 49]. In (autoimmune) ILD, apoptosis and inflammation represent potentially reversible stages of tissue injury [5, 6].

This led us to investigate the diagnostic potential of ^99m^Tc-rhAnnexin V-128 SPECT/CT in two pathophysiologically different models of SSc-ILD. In BLM-challenged and Fra-2 tg mice, the radiotracer ^99m^Tc-rhAnnexin V-128 successfully detected apoptosis in the inflammatory stages as visualized by ex vivo autoradiography and confirmed by biodistribution studies. These results correlated very well with the identification of apoptotic EPC and leukocytes by immunohistochemical fluorescence on tissue level. Most published studies report good correlation between TUNEL-positivity and signal intensity of ^99m^Tc-rhAnnexin V-128 imaging [36, 50–52]. However, although widely accepted as a surrogate marker for apoptosis, TUNEL staining relies on the detection of DNA strand breaks in the nuclei of dead cells [46], which (a) occur after the upregulation of annexin V reflecting later stages of apoptosis [53] and (b) are also characteristic of necrotic cells [54]. In the pathogenesis of ILD, apoptosis rather than necrosis plays a key role in the disease initiation and perpetuation [6]. Thus, we additionally performed staining for cleaved caspase 3, which is a marker for early to mediate processes of apoptosis [53]. The fact that we obtained similar results with both staining methodologies indicates that in our models, the majority of cells were apoptotic. At the tissue level, ^99m^Tc-rhAnnexin V-128 reliably detected apoptosis as assessed by ex vivo autoradiography and biodistribution studies, thereby clearly distinguishing diseased mice from their respective controls. However, in both mouse models, accumulation of the radiotracer and thus the signal intensity in the lungs was too low to allow the diagnosis of ILD in vivo in the tested experimental conditions. Although the most likely explanation is the overall rather low numbers of apoptotic cells in our murine ILD models, we cannot exclude that in our experimental setting the annexin V dose and/or imaging time points were not ideal and might need to be optimized in future trials. However, the number of pulmonary apoptotic cells did not differ from previously published murine ILD studies [39, 43, 55]. In comparison, studies in which ^99m^Tc-rhAnnexin V-128 SPECT/CT had been used to detect acute myocardial infarction, allograft rejection or infectious/septic states in vivo [32, 33, 36, 47] showed substantially higher percentages or amounts of apoptotic cells per tissue area. In general, nuclear imaging of lung pathology compared with solid organs has inherent challenges, especially in small animals with very rapid breathing rates. Ventilation-triggered, i.e. gated, SPECT/CT imaging [56] has great potential to increase both the quality and quantity of SPECT/CT to such an extent that the detection of apoptosis in lung diseases might become possible, at least in disorders with more severe lung damage (e.g. acute toxic or infectious lung injury). Additional improvement might be achieved by excluding signal interference from the unspecific high uptake of the radiotracer in neighboring metabolic organs (kidneys, liver). This might be realized by, for example, focused imaging of the anatomical region of interest, e.g. the chest instead of the whole body, and/or by the adaptation of computational image reconstruction techniques, e.g. by the analysis of defined regions of interest [34–36]. Additionally, ^99m^Tc-rhAnnexin V-128 SPECT/CT has also shown some promise for the monitoring of therapeutic responses and overall disease outcome in, for example, infectious or cardiac diseases [32, 47, 50].

## Conclusions

In conclusion, ^99m^Tc-rhAnnexin V-128 allowed successful visualization of early stages of ILD in two animal models by detection of apoptotic epithelial and/or inflammatory cells in ex vivo samples. However, the transfer of ^99m^Tc-rhAnnexin V-128 SPECT/CT into clinical application to detect early, reversible stages of SSc-ILD remains to be ascertained since in vivo imaging failed to detect lung injury in our two mouse models. Nevertheless, the development of innovative, targeted (nuclear) imaging strategies is currently one of the most challenging prospects in the field of autoimmune diseases to enable the personalized management of these patients.

## Additional files

Additional file 1: Semi-quantification of the number of leucocytes, epithelial cells, myofibroblasts and endothelial cells undergoing apoptosis in the model of BLM-induced lung fibrosis. Co-staining with specific cell markers identified the apoptotic cells (cleaved caspase 3+) as EPC (E-cadherin) and leucocytes (CD45+). Data are expressed as mean ± SD, *n* = 3 (each). (TIF 422 kb)

Additional file 2: Semi-quantification of the number of leucocytes, epithelial cells, myofibroblasts and endothelial cells undergoing apoptosis in the Fra-2 tg mouse model. Co-staining with specific cell markers identified the clear majority of apoptotic cells (cleaved caspase 3+) as leucocytes (CD45+). Data are expressed as mean ± SD, *n* = 3 (each). (TIF 331 kb)

## Abbreviations

% IA/g: Percentage of injected activity per gram tissue
^99m^Tc: Technetium-99 m
ATP: Adenosine triphosphate
BLM: Bleomycin
DAB: 3,3′-Diaminobenzidine
DAMPs: Danger-associated pathogens
EPC: Epithelial cells
Fra-2: Fos-related antigen 2
HE: Hematoxylin and eosin
HMGB-1: High-mobility group protein B1
HPFs: High power fields
HRCT: High-resolution computed tomography
i.v.: Intravenous
ILD: Interstitial lung disease
MBq: MilliBequerel
p.i.: Post injection
PBS: Pphosphate-buffered saline
PET: Positron emission tomography
PFTs: Pulmonary function tests
PS: Phosphatidylserine
RT: Room temperature
SD: Standard deviation
SPECT/CT: Single photon emission computed tomography
SSc: Systemic sclerosis
tg: Transgenic
TGF: Transforming growth factor
TLRs: Toll like-receptors
TUNEL: Terminal deoxynucleotidyl transferase (TdT)-mediated dUTP nick end labeling
vWF: von Willebrand factor
αSMA: Alpha smooth muscle actin
