# Supplementary figures and images for: Evaluation of 99mTc-rhAnnexin V-128 SPECT/CT as a diagnostic tool for early stages of interstitial lung disease associated with systemic sclerosis
Source: Arthritis Res Ther. 2018 Aug 16;20:183. doi: 10.1186/s13075-018-1681-1 (PMC6097327; doi:10.1186/s13075-018-1681-1)

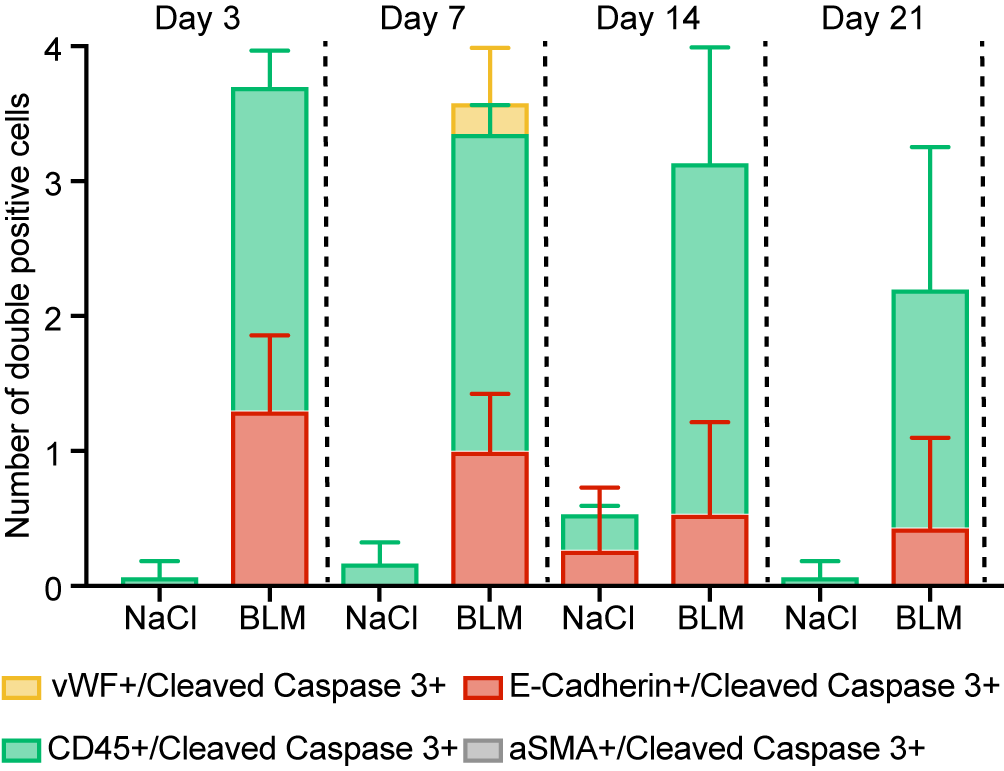

Supplement: Supplementary file 1 — Semi-quantification of the number of leucocytes, epithelial cells, myofibroblasts and endothelial cells undergoing apoptosis in the model of BLM-induced lung fibrosis. Co-staining with specific cell markers identified the apoptotic cells (cleaved caspase 3+) as EPC (E-cadherin) and leucocytes (CD45+). Data are expressed as mean ± SD, n = 3 (each). (TIF 422 kb) [file 13075_2018_1681_MOESM1_ESM.tif]

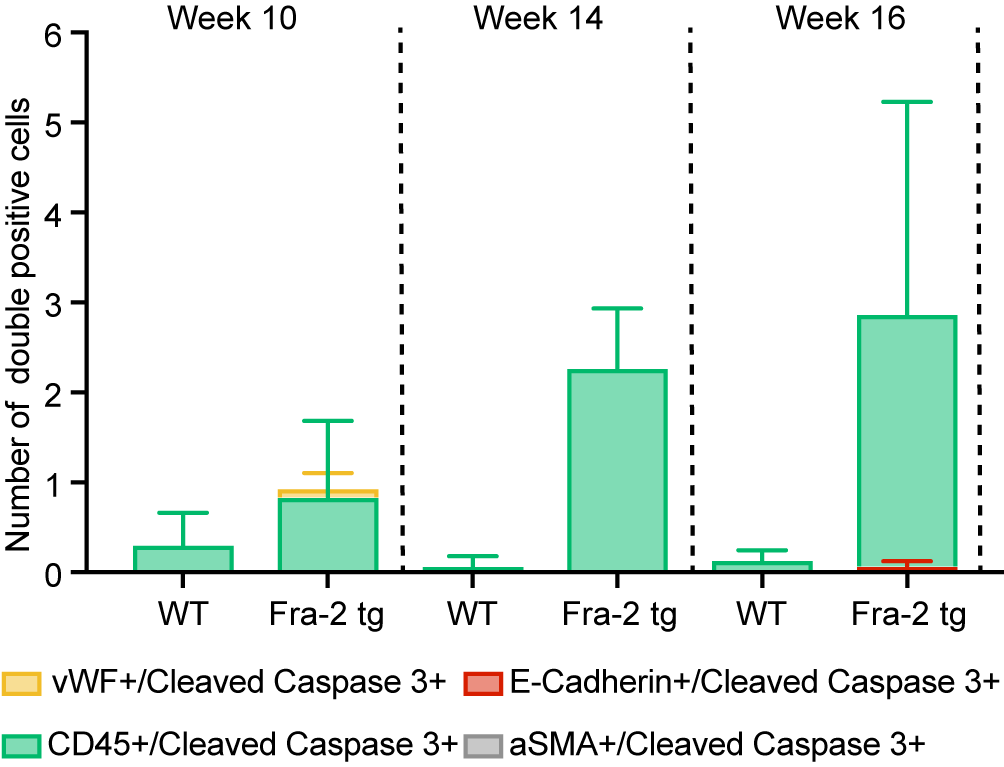

Supplement: Supplementary file 2 — Semi-quantification of the number of leucocytes, epithelial cells, myofibroblasts and endothelial cells undergoing apoptosis in the Fra-2 tg mouse model. Co-staining with specific cell markers identified the clear majority of apoptotic cells (cleaved caspase 3+) as leucocytes (CD45+). Data are expressed as mean ± SD, n = 3 (each). (TIF 331 kb) [file 13075_2018_1681_MOESM2_ESM.tif]
